# Ellagic Acid from *Geranium thunbergii* and Antimalarial Activity of Korean Medicinal Plants

**DOI:** 10.3390/molecules30020359

**Published:** 2025-01-17

**Authors:** Hojong Jun, Joon-Hee Han, Min Hong, Fadhila Fitriana, Jadidan Hada Syahada, Wang-Jong Lee, Ernest Mazigo, Johnsy Mary Louis, Van-Truong Nguyen, Seok Ho Cha, Wanjoo Chun, Won Sun Park, Se Jin Lee, Sunghun Na, Soo-Ung Lee, Eun-Taek Han, Tae-Hyung Kwon, Jin-Hee Han

**Affiliations:** 1 Department of Medical Environmental Biology and Tropical Medicine, School of Medicine, Kangwon National University, Chuncheon 24341, Republic of Korea; goodseed87@gmail.com (H.J.); fadhilafitriana18@gmail.com (F.F.); sjadidanhada@gmail.com (J.H.S.); wjlee@kangwon.ac.kr (W.-J.L.); mazigoernest72@gmail.com (E.M.); johnsymary14@gmail.com (J.M.L.); nguyenvantruong.vmmu@gmail.com (V.-T.N.); ethan@kangwon.ac.kr (E.-T.H.); 2 Institute of Biological Resources, Chuncheon Bioindustry Foundation, Chuncheon 24232, Republic of Korea; cbfhjh@cbf.or.kr (J.-H.H.); fabre_min@cbf.or.kr (M.H.); soounglee@cbf.or.kr (S.-U.L.); 3 Department of Parasitology and Tropical Medicine, Inha University School of Medicine, Incheon 22212, Republic of Korea; shcha@inha.ac.kr; 4 Department of Pharmacology, School of Medicine, Kangwon National University, Chuncheon 24341, Republic of Korea; wchun@kangwon.ac.kr; 5 Department of Physiology, School of Medicine, Kangwon National University, Chuncheon 24341, Republic of Korea; parkws@kangwon.ac.kr; 6 Department of Obstetrics and Gynecology, Kangwon National University Hospital, Chuncheon 24341, Republic of Korea; 23wls@naver.com (S.J.L.); lahun@kangwon.ac.kr (S.N.)

**Keywords:** *Plasmodium falciparum*, malaria, plant extracts, *Geranium thunbergii*, ellagic acid

## Abstract

This study investigates the antimalarial potential of extracts and compounds from various plants used in traditional Korean medicine, in response to the increasing resistance of *Plasmodium falciparum* to standard treatments such as chloroquine and artemisinin. The antimalarial activity screening was conducted on 151 extracts, identifying the top seven candidates, including *Geranium thunbergii* (50% ethanol and 100% methanol extract), *Reynoutria japonica*, *Amomum villosum* (hot water and 50% ethanol extract), *Cinnamomum zeylanicum*, and *Platycodon grandiflorum*. Among these, *G. thunbergii* was identified as the top priority for further analysis due to its high antimalarial activity and high yield of bioactive compounds. The plant extracts were fractionated using ethyl acetate, chloroform, and hot water, and their efficacy against *P. falciparum* was evaluated through IC_50_ determination and microscopic analysis. The compounds evaluated included ellagic acid, gallic acid, afzelin, quercetin, and protocatechuic acid. Among the tested compounds, ellagic acid showed the most potent antimalarial activity with an IC_50_ of 1.60 ± 0.09 µM, followed by gallic acid (39.43 ± 1.48 µM) and afzelin (52.77 ± 1.84 µM). In contrast, quercetin (116.8 ± 3.78 µM) and protocatechuic acid (1.23 ± 0.02 mM) exhibited minimal antimalarial effects. Giemsa staining was employed to visualize parasite morphology and confirmed that ellagic acid is effective in inhibiting growth at the late trophozoite stage. These findings suggest that ellagic acid could serve as a promising lead compound for developing a novel antimalarial agent. This study highlights the importance of exploring plant-based compounds as alternative strategies against drug-resistant malaria. Further investigation into the mechanisms underlying the antimalarial activity of these compounds is necessary to fully validate their therapeutic potential.

## 1. Introduction

Malaria remains a major global health concern, affecting millions of people in 85 endemic countries. An estimated 249 million cases were reported in 2022, resulting in around 608,000 deaths [1]. Additionally, climate change poses new risks by altering the transmission patterns of *Plasmodium* species, including drug-resistant parasites [2]. To overcome this burden, there is an urgent need for effective treatments and sustained efforts to control malaria, especially in the recent emergence of drug-resistant parasites [3].

Historically, malaria treatment has been closely linked to the use of plant-derived compounds. In the early 17th century, people in Peru utilized the bark of the Cinchona tree (*Cinchona* spp.) for the treatment of malaria [4]. This traditional herbal medicine was introduced to Europe, leading to the development of quinine, an alkaloid extracted from Cinchona bark, which became a major agent in antimalarial drugs [5]. Quinine has effectively controlled malaria for decades. However, over time, resistance to these treatment agents began to emerge [3]. By the 20th century, the increasing of resistance to quinine-based therapies, including its synthetic derivatives such as chloroquine (CQ), mefloquine, halofantrine, and lumefantrine was reported in different places, necessitating the need for novel antimalarial drugs [6]. Recently, studies utilizing molecular surveillance through sequencing techniques have revealed significant markers of parasites resistance to CQ. The mutant codon K76T of *Plasmodium falciparum chloroquine resistance transporter* (*pfcrt*) is linked to *P. falciparum* resistance to CQ [7]. The prevalence of the *pfcrt* gene mutant at codon K76T has been reported in different countries including 48.9% in East Africa, 18.6% in South Africa, 58.3% in West Africa, and 80.2% in Asia [7]. Moreover, in vitro studies suggest that *P. falciparum* parasites with the F86Y mutant codon of the *Plasmodium falciparum multi-drug resistance 1* (*pfmdr1*) gene are also resistant to CQ [8]. The *pfmdr1*-86Y mutant codon has been reported elsewhere including 32.4% of cases in East Africa, 36.1% in Southern Africa, 52.2% in West Africa, and 46.4% in Asia [7]. In the 1970s, the novel antimalarial drug artemisinin (ART) was discovered, which is a compound extracted from the Chinese traditional medicinal plant *Artemisia annua*. The introduction of ART helped to overcome chloroquine-resistant malaria parasites [9]. However, recent observations have documented the emergence of resistance to ART in endemic areas, particularly in Africa [10,11,12]. The point mutations in the *Plasmodium falciparum kelch 13* (*pfk13*) gene have been validated as being associated with ART resistance. These mutations have been found in different parts including Southwestern Uganda, Northwestern Tanzania, Rwanda, Ethiopia, and Eritrea [13]. Thus, the World Health Organization (WHO) recommended using artemisinin-based combination therapy (ACT) to slow down the spread of ART drug-resistant parasites.

Considering the limitations of the current antimalarial treatments and the increasing prevalence of drug-resistant parasites, there is a growing global interest in exploring natural products, especially plant extracts, as potential sources of antimalarial agents [14]. Plants have been widely recognized for their medicinal properties in traditional medicine practices across Africa, Latin America, and other regions, serving as a diverse source of natural compounds. Numerous plants used in these traditional practices have demonstrated promising biological activity in preliminary studies, providing strong evidence for further research and validation of their potential as antimalarial agents [15,16,17].

The objective of this study was to screen extracts from the native plants of Korea, many of which have been used in traditional medicine but have not been thoroughly investigated for their potential in malaria treatment. In this study, 151 extracts were screened, and the top seven extracts with antimalarial activity were identified. Among them, *Geranium thunbergii* demonstrated the highest efficacy. A systematic screening approach was then applied to narrow down and identify the active compounds responsible for the antimalarial effects. This research highlights the potential for discovering new plant-based antimalarial compounds that could provide alternatives to the current therapies, especially in regions experiencing resistance to ART or CQ.

## 2. Results

### 2.1. Screening of Plant Extracts for Antimalarial Activity

**Figure 1:**
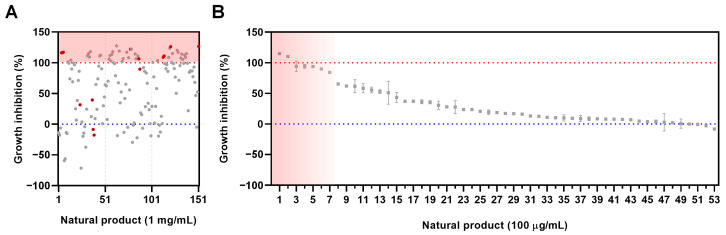
Screening for antimalaria effects of Korean native plant extracts. (**A**) The initial screening was performed on 151 plant extracts at a concentration of 1 mg/mL to assess their growth inhibition activity against *P. falciparum* (3D7). The red circle indicates natural products with hemolytic effect, while the grey circle indicates those without hemolytic effect. The red dashed line represents the positive control (CQ), demonstrating 100% antimalarial activity, while the blue dashed line represents the negative control (1% DMSO). (**B**) The secondary screening was conducted at a concentration of 100 μg/mL on 53 plant extracts that showed higher growth inhibition activity than CQ in *P. falciparum* (3D7) culture. The red background indicates the top seven natural extracts for *P. falciparum* growth inhibition (%). The red dashed line represents the growth inhibition activity of CQ, while the blue dashed line represents the negative control (1% DMSO).

Initially, a total of 151 natural extracts derived from Korean flora were screened and evaluated for antimalarial activity using a growth inhibition assay. The extracts were tested at a concentration of 1 mg/mL in a 96-well microplate format with a 48 h incubation period. CQ served as the positive control, while 1% dimethyl sulfoxide (DMSO) was used as the negative control. The initial screening results revealed that 13 extracts exhibited hemolytic activity at a concentration of 1 mg/mL and were therefore excluded from further testing to avoid interference with the assay. The remaining 138 extracts were assessed for their antimalarial activity, and among these, 53 extracts demonstrated higher antimalarial activity than CQ without causing hemolysis (Figure 1A and Table S1).

In the secondary screening, the 53 active extracts were further evaluated at a 10-fold reduced concentration of 100 µg/mL. The parasite growth inhibition activity showed that seven extracts achieved over 80% growth inhibition (Table 1). Among these, two extracts exhibited greater parasite growth inhibition activity than the CQ control (Figure 1B and Table S2).

### 2.2. Half-Maximal Inhibitory Concentration (IC_50_) of Selected Extracts

Among the top seven active extracts, two were identified as the most potent antimalarial substances. The most effective extract was the 50% ethanol (EtOH) extract from the aerial parts of *Geranium thunbergii*, which exhibited an IC_50_ of 5.31 ± 0.06 µg/mL (Table 1). This was followed by the 100% methanol (MeOH) extract, which demonstrated an IC_50_ of 9.32 ± 2.14 µg/mL (Table 1). These two extracts demonstrated exceptional antimalarial activity, necessitating further fractionation to isolate their active compounds. Additionally, the 100% methanol extract from the roots of *Reynoutria japonica* exhibited an IC_50_ of 16.54 ± 2.52 µg/mL. The hot water extract and the 50% ethanol extract from the fruit of *Amomum villosum* revealed IC_50_ values of 19.11 ± 0.05 µg/mL and 26.66 ± 2.84 µg/mL, respectively (Table 1). Furthermore, the hot water extract of *Cinnamomum zeylanicum* leaf and the 50% ethanol extract of *Platycodon grandiflorum* root revealed IC_50_ values of 35.30 ± 2.04 µg/mL and 64.71 ± 3.19 µg/mL, respectively (Table 1).

### 2.3. Fractionation of Geranium Thunbergii Extracts

The 50% ethanol extract and 100% methanol extract of *G. thunbergii* were fractionated using ethyl acetate (EtOAc), chloroform (CHCl_3_), and hot water to facilitate the identification of their antimalarial potential. Among these fractions, the ethyl acetate fraction from the 50% ethanol extract exhibited the highest parasite growth inhibitory activity, with an IC₅₀ value of 16.38 ± 0.31 µg/mL (Table 2). The chloroform fraction of the same extract demonstrated moderate activity, with an IC_50_ value of 79.21 ± 2.00 µg/mL (Table 2). In contrast, the hot water fraction displayed the weakest activity, with an IC_50_ value of 885.10 ± 9.25 µg/mL (Table 2).

Regarding the 100% methanol extract, the chloroform fraction exhibited the highest antimalarial effect, with an IC_50_ of 27.92 ± 1.69 µg/mL, followed by the ethyl acetate fraction with an IC_50_ of 60.60 ± 1.16 µg/mL. The hot water fraction of the methanol extract showed the lowest antimalarial activity, with an IC_50_ value of 283.5 ± 3.54 µg/mL (Table 2). Overall, the organic solvent fractions (ethyl acetate and chloroform) demonstrated greater bioactivity for parasite growth inhibition than the aqueous fractions.

### 2.4. Analysis of Chemical Composition and Extraction Yield

**Figure 2:**
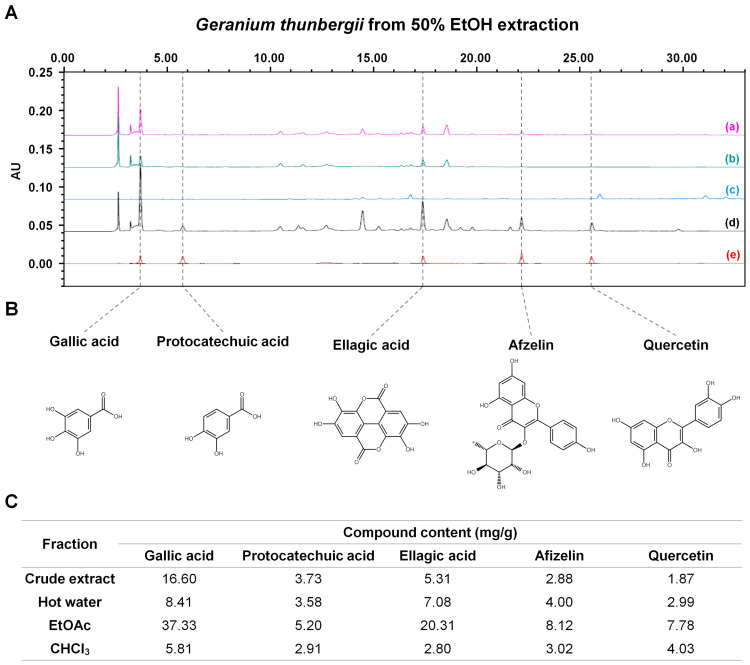
HPLC analysis and major contents of *Geranium thunbergii* extracts. (**A**) HPLC chromatogram of various fractions of *G. thunbergii* from 50% EtOH extraction: (a) *G. thunbergii* crude extract, (b) hot water fraction, (c) chloroform (CHCl_3_) fraction, (d) ethyl acetate (EtOAc) fraction, (e) mixed standard compounds. (**B**) Structures of major compounds from *G. thunbergii*. (**C**) Content of each natural compound (mg/g) from various fractions.

Following the fractionation of *G. thunbergii* extracts, High-Performance Liquid Chromatography (HPLC) was employed to measure the bioactive compounds associated with the observed antimalarial activity. The ethyl acetate, chloroform, and hot water fractions were analyzed to quantify major compounds (Figure 2A). The extraction yield of *G. thunbergii* using 50% ethanol was measured at 18.9% and was subsequently utilized for fractionation. The yields of the individual fractions were as follows: chloroform fraction (4.34%), ethyl acetate fraction (9.11%), and DDW fraction (76.99%). Subsequently, the extract and each fraction were analyzed for the content of five major compounds (Figure 2A,B). The HPLC analysis identified several known compounds, including gallic acid, protocatechuic acid, ellagic acid, afzelin, and quercetin (Figure 2B). The ethyl acetate fraction of *G. thunbergii* exhibited the highest concentrations of the measured compounds, specifically containing gallic acid at 37.33 mg/g, protocatechuic acid at 5.20 mg/g, ellagic acid at 20.31 mg/g, afzelin at 8.12 mg/g, and quercetin at 7.78 mg/g (Figure 2C). In contrast, the chloroform fraction displayed significantly lower concentrations, with gallic acid at 5.81 mg/g, protocatechuic acid at 2.91 mg/g, ellagic acid at 2.80 mg/g, afzelin at 3.02 mg/g, and quercetin at 4.03 mg/g (Figure 2C). Similarly, the hot water fraction also contained reduced levels of these compounds, including gallic acid at 8.41 mg/g, protocatechuic acid at 3.58 mg/g, ellagic acid at 7.08 mg/g, quercetin at 2.99 mg/g, and afzelin at 4.00 mg/g (Figure 2C).

### 2.5. Growth Inhibition Activity of Bioactive Compounds

**Figure 3:**
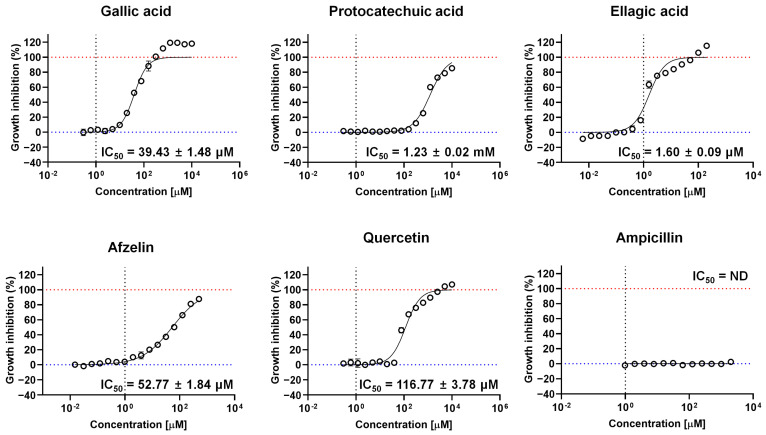
The IC_50_ values for *Plasmodium falciparum* growth inhibition were determined for the major compounds in the ethyl acetate fraction of *Geranium thunbergii*. The compounds tested included gallic acid, protocatechuic acid, ellagic acid, afzelin, and quercetin. Ampicillin was used as the experimental negative control. The red dashed line indicates the positive control (CQ), which is set at 100% growth inhibition activity, while the blue dashed line represents the negative control (1% DMSO).

The five measured compounds (gallic acid, protocatechuic acid, ellagic acid, afizelin, and quercetin) were individually evaluated for their growth inhibition activity against *P. falciparum*. Among these compounds, ellagic acid exhibited the highest inhibitory activity, with an IC_50_ value of 1.60 ± 0.09 µM (Figure 3). This was followed by gallic acid (IC_50_ of 39.43 ± 1.48 µM) and afzelin (IC_50_ of 52.77 ± 1.84 µM). In contrast, quercetin and protocatechuic acid displayed minimal antimalarial effects, with IC_50_ values of 116.77 ± 3.78 µM and 1.23 ± 0.02 mM, respectively (Figure 3).

**Figure 4:**
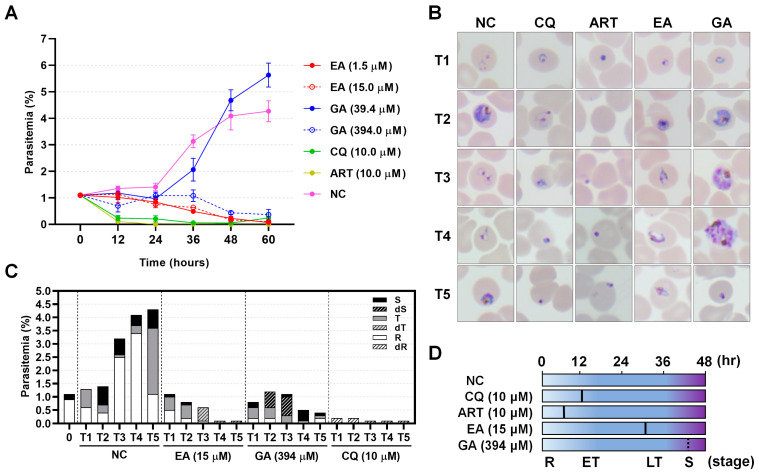
*Plasmodium falciparum* growth inhibition by ellagic acid (EA) and gallic acid (GA). (**A**) Parasitemia in *P. falciparum* treated with EA and GA was monitored at sequential time points by analyzing Giemsa-stained blood smears. Both compounds were tested at their IC_50_ concentrations and at 10-fold higher concentrations to assess parasite morphology upon death. EA, ellagic acid; GA, gallic acid; CQ, chloroquine; ART, artemisinin; NC, negative control which contained 0.5% DMSO. (**B**) Giemsa-stained thin smears were used to identify the predominant developmental stage and morphology of the parasites. (**C**) Proportions of parasite stages after treatment with high concentrations of EA (15 µM) and GA (394 µM) were recorded. dR, dead ring; R, viable ring; dT, dead trophozoite; T, viable trophozoite; dS, dead schizont; S, viable schizont. (**D**) Estimation of the effective time and stage of growth inhibition in response to high concentrations of EA and GA. R, ring; ET, early trophozoite; LT, late trophozoite; S, schizont stage.

To confirm growth inhibition activity and the specific developmental stages affected, parasite cultures were monitored at sequential time points. Ellagic acid (EA) and gallic acid (GA) were tested at both IC_50_ and 10-fold higher concentrations to assess the morphology of live and dead parasites. Overall, parasitemia decreased in parasites treated with ellagic acid, CQ, and ART (Figure 4A). CQ and ART are fast-acting drugs that quickly decrease visible live parasites within 12 h under microscopy. However, ellagic acid showed no significant difference in parasitemia compared to the non-treated control at 12 h and acted more slowly, showing a significant decrease in parasitemia at 24 h post-treatment (Figure 4A and Figure S1). In contrast, gallic acid at low concentrations initially promoted parasite survival beyond 36 h (Figure 4A). However, at higher concentrations of gallic acid (394 µM), significant inhibition of *P. falciparum* growth was observed (Figure 4A and Figure S1). At each time point, ellagic acid (15 µM) halted parasite development at the trophozoite stage (Figure 4B,C). While gallic acid (394 µM) allowed parasite development to the schizont stage, though at a delayed development time, with most parasites failing to invade new erythrocytes (Figure 4B,C). Morphologically and based on the proportion of developmental stage identification, parasites treated with CQ showed dormant rings or dead at early trophozoite stages, and ART-treated cultures had dormant ring stages (Figure 4D). On the other hand, ellagic acid arrested parasites development at the mid-to-late trophozoite stage, while gallic acid produced unhealthy schizonts (Figure 4D).

## 3. Discussion

This study demonstrates the systematic investigation of natural products as a source of novel antimalarial agents by utilizing a structured approach that starts with a comprehensive library of native Korean plants extracts and gradually narrows down to the most active fractions and compounds. This approach enables researchers to efficiently identify promising candidates for further studies [18]. In response to the increasing resistance to conventional antimalarial drugs, this study emphasizes the potential of compounds derived from native Korean plants in discovering novel antimalarial drugs. Korea is a *Plasmodium vivax* endemic country, yet it has not been extensively explored in antimalarial plant research [19]. In this study, the extracts of *Geranium thunbergii*, *Reynoutria japonica*, *Amomum villosum*, *Cinnamomum zeylanicum*, and *Platycodon grandiflorumi* demonstrated potential antimalarial effects on *P. falciparum*. Among them, *G. thunbergii* exhibited the most active antimalarial activity against the *P. falciparum* 3D7 strain. This plant has been used for centuries in traditional Korean medicine to treat stomach diseases including dysentery and stomach ulcers [20,21]. Additionally, the ethyl acetate fraction of *G. thunbergii* has previously shown antioxidant and antimicrobial activities [22].

Among the compounds analyzed in this study, ellagic acid exhibited the most bioactivity for growth inhibitory effects on *P. falciparum*. Ellagic acid is a natural polyphenolic compound found in many fruits, nuts, and green tea, and it has been reported to possess multiple pharmacological activities [23]. Phenolic compounds demonstrate antimicrobial activities through various mechanisms. One key mechanism is their ability to dissociate at a physiological pH, creating a proton gradient that disrupts membrane potential and calcium signaling [24]. In malaria parasites, calcium signaling is crucial for the survival and invasion of new host cells [25]. The disruption of membrane potential and calcium signaling can impair the function of essential ion pumps, sequester critical metal ions, and inactivate membrane proteins involved in nutrient uptake [24]. Interestingly, ellagic acid has shown a strong potential as a therapeutic agent for hemoparasites, including not only *Plasmodium* but also *Babesia* and *Theileria* [26]. The mode of action of ellagic acid in *P. falciparum* may involve altering the food vacuole pH by inhibiting the regulation of proton pumps by acidification on the membrane [27]. Additionally, ellagic acid has been suggested as a useful bioflavonoid for alleviating severe malaria pathogenesis by reducing cytokine storms and oxidative stress [28]. In this study, ellagic acid was identified as the primary active compound in the *G. thunbergii* extract, with an IC_50_ of 1.60 ± 0.09 µM against *P. falciparum* (3D7 strain). A previous report demonstrated a significantly lower IC_50_ (105–330 nM) in drug-resistant *P. falciparum* strains, including F32, Dd2, FcB1, W2, and FcM29 [29]. Although the source of ellagic acid differed between the two studies, the purity of compounds was similar. These findings suggest that ellagic acid may exhibit greater susceptibility in drug-resistant *P. falciparum* strains, particularly those resistant to CQ and mefloquine. Moreover, prior research has demonstrated that ellagic acid is an effective agent against *P. falciparum* and exerts a synergistic effect when combined with other drugs such as ART and atovaquone [26,29,30]. Artemisinin acts quickly, typically within 5 h of administration, and the remaining parasites could potentially be cleared by ellagic acid, as it effectively arrests development at the mid-to-late trophozoite stage. Thus, it is necessary to consider developing ellagic acid derivatives to enhance safety and anti-hemoparasitic activity.

Additionally, gallic acid is well known for its antioxidant properties and has demonstrated moderate antimalarial efficacy in previous studies [31]. It is hypothesized that gallic acid exerts its effects by inducing oxidative stress in the parasite, which disrupts its metabolic processes and ultimately inhibits growth [32]. Although it showed lower IC_50_ values than ellagic acid, gallic acid remains a promising candidate for use in combination therapies or as part of a multi-compound strategy for malaria treatment.

The significance of *G. thunbergii* in this study is shown by the yields of 20.31 mg/g of ellagic acid and 36.33 mg/g of gallic acid in the ethyl acetate fraction, which are higher yields compared to those of other plant species. For instance, *Rubus fruticosus* (blackberry) has reported ellagic acid yields ranging from 1.19 mg/g to 3.23 mg/g, depending on the cultivar, which equates to 1.2–3.2 mg/g dry weight [33]. Similarly, *Punica granatum* (pomegranate) has shown ellagic acid yields between 13.4 mg/g and 23.8 mg/g, depending on the processing method [34]. These comparisons highlight the abundance of ellagic acid in *G. thunbergii*. In terms of gallic acid, *Terminalia chebula* (myrobalan) yielded 18.52 mg/g [35], which is considerably lower than the 36.33 mg/g observed in *G. thunbergii*. The higher concentrations of these compounds indicate that *G. thunbergii* is a potent source of bioactive compounds, supporting its potential for antimalarial activity. These findings underscore the importance of exploring traditional medicinal plants for their pharmacological potential. Optimizing extraction techniques and further characterizing bioactive compounds may contribute to the development of new therapeutic agents.

## 4. Materials and Methods

### 4.1. Extraction Methods for Natural Products and Geranium thunbergii

A total of 151 natural extracts derived from Korean flora (55 plant species) were extracted using 50% ethanol, 100% methanol, and hot water, as described elsewhere (Table S1) [22]. Briefly, *G. thunbergii* extracts were prepared using either 50% ethanol or 100% methanol. Dried *G. thunbergii* was ground into a fine powder using a grinder (Daesung Artlon, Paju, Korea) and then sieved to achieve an 80-mesh size (Figure S1). For the extraction process, 100 g of the powdered material was combined with 1 L of either 50% ethanol or 100% methanol (Sigma-Aldrich, St. Louis, MO, USA) and mixed at room temperature for 6 h. The mixture was then filtered through Whatman No. 2 filter paper. The resulting filtrate was concentrated under reduced pressure using a rotary evaporator (Rotavapor, Buchi Labortechnik AG, Flawil, Switzerland) and subsequently freeze-dried with a lyophilizer (Ilshinbiobase Co., Ltd., Yangju, Republic of Korea) to obtain the final extract for further analysis.

### 4.2. Fractionation and Antimalarial Activity Experiment Using HPLC in Geranium thunbergii

#### 4.2.1. Fractionation

To identify the active compounds responsible for antimalarial activity in *G. thunbergii* extracts, fractionation was performed on the most potent extracts from both 50% ethanol and 100% methanol extractions. The fractionation process involved three solvents: water, ethyl acetate, and chloroform, using a 5 L separatory funnel. A total of 10 g of freeze-dried extract was dissolved in distilled water and sequentially partitioned with 1 L each of ethyl acetate and chloroform. This procedure yielded three distinct fractions: aqueous, ethyl acetate, and chloroform. Each fraction was filtered and concentrated under reduced pressure using a rotary evaporator.

#### 4.2.2. High-Performance Liquid Chromatography (HPLC) Analysis

The extracted samples were prepared by dissolving them in 70% ethanol at a concentration of 10 mg/mL. The solution was sonicated for 2 h and then filtered through a 0.45 μm syringe filter prior to analysis. Standard compounds, including ellagic acid (≥98%, Sigma-Aldrich, MO, USA), gallic acid (≥99%, Sigma-Aldrich), protocatechuic acid (≥98%, Sigma-Aldrich), afzelin (≥95%, Sigma-Aldrich), and quercetin (≥95%, Sigma-Aldrich) were diluted in ethanol for comparison. The analysis was performed using an HPLC system (Arc HPLC system, Waters, Milford, MA, USA) equipped with a reverse-phase column (Agilent, Santa Clara, CA, USA) ZORBAX Eclipse Plus C18, 4.6 mm × 250 mm, 5 μm) at 35 °C. Detection occurred at 206 nm using a UV detector. The mobile phase, composed of water (A) and acetonitrile (B), was run in gradient mode at a flow rate of 1 mL/min as follows: 76% B from 0 to 4 min, 83% B from 4 to 12 min, and 95% B from 12 to 17 min, maintaining 95% B until 20 min, then returning to 76% B until the end of the 30 min run.

### 4.3. In Vitro Culture of Plasmodium falciparum

The *P. falciparum* (3D7 strain; ATCC) was cultured with human erythrocytes at a 2% hematocrit in RPMI-1640 medium (Gibco, Medford, MA, USA). The culture medium was supplemented with 2.3 g/L sodium bicarbonate (Amresco, Dallas, TX, USA), 0.05 g/L hypoxanthine (Sigma-Aldrich, Burlington, MA, USA), 10% Albumax I (Gibco), and 10 mg/mL gentamicin (Gibco). The culture was maintained under an atmosphere of 90% N_2_, 5% O_2_, and 5% CO_2,_ and incubated at 37 °C.

### 4.4. Growth Inhibition Assay for Screening

For primary screening, the antimalarial activity of 151 natural extracts was assessed using the SYBR Green I (Invitrogen, Waltham, MA, USA)-based growth inhibition assay [36]. Briefly, each extract was prepared at a concentration of 1 mg/mL and applied in duplicate to 96-well plates. Each well was inoculated with 100 µL of *P. falciparum* 3D7 culture at 1% parasitemia and 2% hematocrit, followed by a 48 h incubation. CQ (10 µM) was used as a positive control, while uninfected and infected erythrocytes treated with 1% DMSO served as negative controls. After the 48 h treatment with the natural extracts, each well received 4X lysis buffer (80 mM Tris-HCl, 20 mM EDTA, 0.032% saponin, and 0.32% Triton X-100 in distilled deionized water) to lyse the erythrocytes and release the parasitic DNA for detection. SYBR Green I (2500X) was added to the buffer, facilitating fluorescence-based detection. The plates were then shaken for five minutes, and fluorescence was measured using a microplate reader (Molecular Devices, San Jose, CA, USA) with excitation at 485 nm and emission at 530 nm.

Secondary screening was conducted on the active extracts identified in the primary screening. These extracts were tested at a concentration of 100 µg/mL under the same conditions used in the primary screen.

### 4.5. Half-Maximal Inhibitory Concentration (IC_50_) Determination

To determine the IC_50_ values, the top seven active extracts were serially diluted in 2-fold increments from 400 µg/mL to 0.2 µg/mL. Similarly, the fractions were diluted in the range of 1 mg/mL to 0.2 µg/mL, while the compounds were prepared in concentrations ranging from 10 mM to 0.3 µM. All experiments were performed in triplicate. The IC_50_ values for each extract and compound were calculated based on the fluorescence measurements obtained from the SYBR Green I-based growth inhibition assay as described above. To evaluate the effect of parasite growth inhibition, the activity was compared to a negative control group (1% DMSO) and a positive control group (CQ). To ensure data comparability, the mean fluorescence intensities for both the control and natural extract-treated groups were calculated and expressed as a percentage of the control group, which was set at 100%. This percentage reflects the extent of parasite growth inhibition achieved by the treatment of natural extract. The percentage of growth inhibition was calculated using the following formula: Growth inhibition of parasite (%) = (1 − (Test well − Average of positive control)/(Average of negative control − Average of positive control)) × 100. This analysis was conducted using Excel software (Microsoft Inc., Washington, DC, USA). The results were presented graphically as scatter plots to identify primary hit compounds and as log dose–response curves for calculating IC_50_ values. The IC_50_ values were analyzed using a linear regression curve fit in GraphPad Prism v8.

### 4.6. Microscopic Examination

The IC_50_ concentrations and 10-fold higher doses of ellagic acid and gallic acid were validated through microscopic examination to assess the morphology and developmental stages of *P. falciparum*. Efficacy tests for each compound were conducted in triplicate, using 100 µL of parasite culture per well, as described above. The IC_50_ values were determined to be 1.60 µM for ellagic acid and 39.43 µM for gallic acid. These concentrations were applied to *P. falciparum* cultures over a 60 h period, with samples collected and Giemsa-stained at 12 h intervals to evaluate the effects on the parasites microscopically.

### 4.7. Statistical Analysis

Statistical analysis of parasitemia between compound-treated and non-treated groups was conducted using an unpaired *t*-test, with significance indicated by three asterisks (*p* < 0.001), two asterisks (*p* < 0.01), and one asterisk (*p* < 0.05).

## 5. Conclusions

This study highlights the significant potential of native Korean plants, particularly *G*. *thunbergii*, as a valuable source of novel antimalarial agents. Through a systematic analysis, several plant extracts were evaluated for their antimalarial efficacy, among which *G*. *thunbergii* emerged as the most promising candidate. The most potent bioactive compound, ellagic acid, demonstrated significant growth-inhibitory effects against *P*. *falciparum*. Additionally, the high yields of ellagic acid and gallic acid in the ethyl acetate fraction of *G. thunbergii* suggest its high potential for large-scale applications in drug discovery. These results emphasize the importance of investigating traditional medicinal plants for the development of antimalarial drugs, alongside the optimization of extraction methods and the further characterization of their bioactive compounds. Ongoing research into the synergistic effects of these compounds with existing antimalarial therapies may facilitate the development of more effective combination treatments for malaria.

## Figures and Tables

**Table 1:** IC₅₀ of the top 7 plant crude extracts for *Plasmodium falciparum* growth inhibition.

Rank	Plant Species	Source	Extraction Solvent	IC_50_ ± SD (μg/mL)
1	*Geranium thunbergia*	Aerial parts	50% EtOH	5.31 ± 0.06
2	*Geranium thunbergia*	Aerial parts	MeOH	9.32 ± 2.14
3	*Reynoutria japonica*	Root	MeOH	16.54 ± 2.52
4	*Amomum villosum*	Fruit	Hot water	19.11 ± 0.05
5	*Amomum villosum*	Fruit	50% EtOH	26.66 ± 2.84
6	*Cinnamomum zeylanicum*	Leaf	Hot water	35.30 ± 2.04
7	*Platycodon grandiflorum*	Root	50% EtOH	64.71 ± 3.19

**Table 2:** IC₅₀ of *Geranium thunbergii* fraction from 50% EtOH and MeOH extraction for *Plasmodium falciparum* growth inhibition.

Rank	Extraction	IC_50_ ± SD (μg/mL) of Each Fraction		
		Hot Water	EtOAc	CHCl_3_
1	50% EtOH	885.10 ± 9.25	16.38 ± 0.31	79.21 ± 2.00
2	MeOH	283.50 ± 3.54	60.60 ± 1.16	27.92 ± 1.69

## Data Availability

Data are contained within the article and Supplementary Materials.

## Supplementary Materials

The following supporting information can be downloaded at: https://www.mdpi.com/article/10.3390/molecules30020359/s1, Table S1. The list of 151 natural extracts for initial screening (1 mg/mL) and their *P. falciparum* growth inhibition activity (%). Table S2. The list of 53 natural extracts for secondary screening (100 μg/mL) and their *P. falciparum* growth inhibition activity (%). Figure S1. Dried *Geranium thunbergii* which is used for extraction.
