# Supplementary material for: Ellagic Acid from Geranium thunbergii and Antimalarial Activity of Korean Medicinal Plants
Source: Molecules. 2025 Jan 17;30(2):359. doi: 10.3390/molecules30020359 (PMC11767465; doi:10.3390/molecules30020359)
Supplement: Supplementary file 1 [file molecules-30-00359-s001.zip › molecules-3398651-supplementary.pdf]

Supplementary Materials

Ellagic Acid from *Geranium thunbergii* and Antimalarial Activity of Korean Medicinal Plants

Hojong Jun [1,†], Joon-Hee Han [2,†], Min Hong [2], Fadhila Fitriana [1], Jadidan Hada Syahada [1], Wang-Jong Lee [1], Ernest Mazigo [1], Johnsy Mary Louis [1], Van-Truong Nguyen [1], Seok Ho Cha [3], Wanjoo Chun [4], Won Sun Park [5], Se Jin Lee [6], Sunghun Na [6], Soo-Ung Lee [2], Eun-Taek Han [1], Tae-Hyung Kwon [2,*] and Jin-Hee Han [1,*]

[1] Department of Medical Environmental Biology and Tropical Medicine, School of Medicine, Kangwon National University, Chuncheon 24341, Republic of Korea; goodseed87@gmail.com (H.J.); fadhilafitriana18@gmail.com (F.F.); sjadidanhada@gmail.com (J.H.S.); wjlee@kangwon.ac.kr (W.-J.L.); mazigoernest72@gmail.com (E.M.); johnsymary14@gmail.com (J.M.L.); nguyenvantruong.vmmu@gmail.com (V.-T.N.); ethan@kangwon.ac.kr (E.-T.H.)

[2] Institute of Biological Resources, Chuncheon Bioindustry Foundation, Chuncheon 24232, Republic of Korea; cbfhjh@cbf.or.kr (J.-H.H.); fabre_min@cbf.or.kr (M.H.); soounglee@cbf.or.kr (S.-U.L.)

[3] Department of Parasitology and Tropical Medicine, Inha University School of Medicine, Incheon 22212, Republic of Korea; shcha@inha.ac.kr

[4] Department of Pharmacology, School of Medicine, Kangwon National University, Chuncheon 24341, Republic of Korea; wchun@kangwon.ac.kr

[5] Department of Physiology, School of Medicine, Kangwon National University, Chuncheon 24341, Republic of Korea; parkws@kangwon.ac.kr

[6] Department of Obstetrics and Gynecology, Kangwon National University Hospital, Chuncheon 24341, Republic of Korea; 23wls@naver.com (S.J.L.); lahun@kangwon.ac.kr (S.N.)

* Correspondence: team0218@cbf.or.kr (T.-H.K.); han.han@kangwon.ac.kr (J.-H.H.)

† These authors contributed equally to this work.

Table S1. The list of 151 natural extracts for initial screening (1 mg/mL) and their *P. falciparum* growth inhibition activity (%).

No.	Plant species	Extraction Solvent	*P. falciparum* growth inhibition (%)		Hemolysis effect
			Mean	SD	
1	*Allium tuberosum Rottler*	Hot water	-13.87	4.80	
2	*Allium tuberosum Rottler*	50% EtOH	-17.72	8.86	
3	*Allium tuberosum Rottler*	100% MeOH	-6.28	1.27	
4	*Terminalia chebula Retzius*	Hot water	116.05	0.24	○
5	*Terminalia chebula Retzius*	50% EtOH	116.62	0.23	○
6	*Terminalia chebula Retzius*	100% MeOH	116.99	1.11	○
7	*Pueraria lobata Ohwi*	Hot water	-59.42	0.53	
8	*Pueraria lobata Ohwi*	50% EtOH	-56.35	0.86	
9	*Pueraria lobata Ohwi*	100% MeOH	-13.58	9.36	
10	*Pueraria lobata Ohwi*	Hot water	-15.19	4.08	
11	*Pueraria lobata Ohwi*	50% EtOH	101.15	2.07	
12	*Pueraria lobata Ohwi*	100% MeOH	99.18	0.59	
13	*Chrysanthemum indicum L.*	50% EtOH	73.69	0.16	
14	*Chrysanthemum indicum L.*	100% MeOH	104.15	0.17	
15	*Glycyrrhiza uralensis Fischer*	Hot water	89.68	1.62	
16	*Glycyrrhiza uralensis Fischer*	50% EtOH	68.96	4.39	
17	*Glycyrrhiza uralensis Fischer*	100% MeOH	99.12	0.68	
18	*Angelica koreana L.*	Hot water	96.51	0.73	
19	*Angelica koreana L.*	50% EtOH	25.01	7.22	
20	*Angelica koreana L.*	100% MeOH	6.59	2.32	
21	*Zingiber officinale Roscoe*	Hot water	38.51	2.85	
22	*Zingiber officinale Roscoe*	50% EtOH	71.25	2.33	
23	*Zingiber officinale Roscoe*	100% MeOH	86.08	3.19	○
24	*Castanea crenata Siebold & Zucc.*	Hot water	31.59	7.88	
25	*Castanea crenata Siebold & Zucc.*	50% EtOH	-71.40	25.58	
26	*Castanea crenata Siebold & Zucc.*	100% MeOH	3.67	4.31	
27	*Rehmannia glutinosa (Gaertn.) Libosch. ex Steud.*	Hot water	-19.80	3.71	
28	*Rehmannia glutinosa (Gaertn.) Libosch. ex Steud.*	50% EtOH	-14.61	8.35	
29	*Rehmannia glutinosa (Gaertn.) Libosch. ex Steud.*	100% MeOH	-3.99	1.35	
30	*Gallus domesticus Brisson*	Hot water	-37.51	1.34	
31	*Gallus domesticus Brisson*	50% EtOH	24.35	2.82	
32	*Gallus domesticus Brisson*	100% MeOH	111.16	6.79	
33	*Cinnamomum zeylanicum Blume(leaf)*	Hot water	114.98	0.36	
34	*Cinnamomum zeylanicum Blume(leaf)*	50% EtOH	109.32	3.08	
35	*Cinnamomum zeylanicum Blume(twig)*	Hot water	117.95	1.61	

36	*Cinnamomum zeylanicum Blume(twig)*	100% MeOH	12.40	10.56	
37	*Spatholobus suberectus Dunn*	Hot water	39.35	0.73	
38	*Spatholobus suberectus Dunn*	50% EtOH	-8.55	8.84	
39	*Spatholobus suberectus Dunn*	100% MeOH	-17.87	4.82	
40	*Ligusticumm tenuissimum NAKAI KITAG.[KHP]*	Hot water	9.13	2.58	
41	*Ligusticumm tenuissimum NAKAI KITAG.[KHP]*	50% EtOH	64.58	1.08	
42	*Ligusticumm tenuissimum NAKAI KITAG.[KHP]*	100% MeOH	24.24	0.53	
43	*Sophora flavescens*	Hot water	73.83	0.35	
44	*Sophora flavescens*	50% EtOH	94.37	0.29	
45	*Sophora flavescens*	100% MeOH	110.99	0.07	
46	*Viscum album var. coloratum (Kom.) Ohwi*	Hot water	112.17	1.04	
47	*Viscum album var. coloratum (Kom.) Ohwi*	50% EtOH	90.08	7.74	
48	*Viscum album var. coloratum (Kom.) Ohwi*	100% MeOH	0.85	7.96	
49	*Caragana chamlagu Lam.*	Hot water	29.91	2.22	
50	*Caragana chamlagu Lam.*	50% EtOH	44.24	0.80	
51	*Caragana chamlagu Lam.*	100% MeOH	64.20	1.70	
52	*Drynaria fortunei Smith*	Hot water	62.34	2.30	
53	*Drynaria fortunei Smith*	50% EtOH	7.57	10.92	
54	*Drynaria fortunei Smith*	100% MeOH	69.77	2.65	
55	*Amomum villosum*	Hot water	105.88	1.40	
56	*Amomum villosum*	50% EtOH	111.69	1.60	
57	*Amomum villosum*	100% MeOH	102.71	0.30	
58	*Agastache rugosa O. Kuntze*	Hot water	113.49	0.17	
59	*Agastache rugosa O. Kuntze*	50% EtOH	117.86	1.80	
60	*Agastache rugosa O. Kuntze*	100% MeOH	99.79	8.26	
61	*Tussilago farfara L.*	Hot water	98.52	6.30	
62	*Tussilago farfara L.*	50% EtOH	78.90	0.10	
63	*Tussilago farfara L.*	100% MeOH	127.30	1.45	
64	*Trichosanthes kirilowii Maximowicz*	Hot water	-18.58	1.48	
65	*Dianthus sinensis L.*	Hot water	67.38	3.82	
66	*Dianthus sinensis L.*	50% EtOH	118.93	9.08	
67	*Pericaeta communisma Gate et Hatai*	Hot water	34.59	11.06	
68	*Pericaeta communisma Gate et Hatai*	50% EtOH	52.38	6.57	
69	*Cibotium barometz J. Smith*	Hot water	101.70	5.02	
70	*Cibotium barometz J. Smith*	50% EtOH	106.43	2.39	
71	*Chinemys reevesii Gray*	Hot water	-13.22	1.61	
72	*Chinemys reevesii Gray*	50% EtOH	41.85	3.86	
73	*Selaginella tamariscina Spring*	Hot water	64.97	5.39	
74	*Selaginella tamariscina Spring*	50% EtOH	117.14	0.04	
75	*Selaginella tamariscina Spring*	100% MeOH	-5.01	4.29	

76	*Rosa laevigata Michaux*	Hot water	108.16	0.93	
77	*Rosa laevigata Michaux*	50% EtOH	55.65	3.70	
78	*Lonicera japonica Thunberg*	Hot water	122.01	1.39	○
79	*Lonicera japonica Thunberg*	50% EtOH	122.08	1.69	
80	*Panicum miliaceum L.*	Hot water	-29.50	11.91	
81	*Panicum miliaceum L.*	50% EtOH	-1.60	13.82	
82	*Platycodon grandiflorum*	Hot water	2.14	1.65	
83	*Platycodon grandiflorum*	50% EtOH	114.31	1.00	
84	*Platycodon grandiflorum*	100% MeOH	106.20	1.10	
85	*Raphanus sativus L.*	Hot water	-22.16	1.03	
86	*Echinops setifer Iljin*	Hot water	110.98	1.39	
87	*Echinops setifer Iljin*	50% EtOH	106.26	10.62	○
88	*Echinops setifer Iljin*	100% MeOH	89.56	3.66	○
89	*Phragmites communis Trin.*	Hot water	18.54	4.07	
90	*Phragmites communis Trin.*	50% EtOH	15.49	12.01	
91	*Phragmites communis Trin.*	100% MeOH	-6.56	15.91	
92	Vespae Nidus	Hot water	92.04	1.48	
93	Vespae Nidus	50% EtOH	86.04	4.24	
94	Vespae Nidus	100% MeOH	28.54	12.49	
95	*Salvia miltiorrhiza Bunge*	Hot water	83.20	0.76	
96	*Salvia miltiorrhiza Bunge*	50% EtOH	66.47	3.15	
97	*Salvia miltiorrhiza Bunge*	100% MeOH	34.06	1.10	
98	*Angelica gigas Nakai*	Hot water	23.52	3.99	
99	*Angelica gigas Nakai*	50% EtOH	0.80	12.17	
100	*Angelica gigas Nakai*	100% MeOH	-19.10	1.95	
101	*Cirsium japonicum var. ussuriense (Regel) Kitam*	Hot water	28.96	3.07	
102	*Cirsium japonicum var. ussuriense (Regel) Kitam*	50% EtOH	100.35	2.97	
103	*Cirsium japonicum var. ussuriense (Regel) Kitam*	100% MeOH	77.01	8.46	
104	*Areca catechu L.*	Hot water	1.96	16.10	
105	*Areca catechu L.*	50% EtOH	28.97	5.83	
106	*Illicum verum*	Hot water	57.94	7.23	
107	*Illicum verum*	50% EtOH	103.45	2.88	
108	*Prunus persica Batsch*	Hot water	-19.39	2.50	
109	*Prunus persica Batsch*	50% EtOH	-1.86	4.16	
110	*Prunus persica Batsch*	100% MeOH	-12.80	0.00	
111	*Aralia cordata*	Hot water	12.76	3.53	
112	*Aralia cordata*	50% EtOH	1.00	16.06	
113	*Prunella vulgaris L.*	Hot water	108.81	1.98	○
114	*Prunella vulgaris L.*	50% EtOH	111.03	1.03	○
115	*Prunella vulgaris L.*	100% MeOH	98.25	0.44	

116	*Nelumbo nucifera Gaertner*	Hot water	90.24	2.52	
117	*Nelumbo nucifera Gaertner*	50% EtOH	96.71	3.47	
118	*Nelumbo nucifera Gaertner*	100% MeOH	85.81	1.44	
119	*Eclipta prostrata L.*	Hot water	108.79	4.93	
120	*Eclipta prostrata L.*	50% EtOH	123.56	1.56	
121	*Eclipta prostrata L.*	100% MeOH	126.21	0.14	
122	*Albizzia julibrissin Durazz.*	Hot water	100.20	0.82	
123	*Albizzia julibrissin Durazz.*	50% EtOH	114.89	0.20	
124	*Albizzia julibrissin Durazz.*	100% MeOH	98.42	0.13	
125	*Rosa rugosa Thunberg.*	Hot water	90.46	0.59	
126	*Rosa rugosa Thunberg.*	50% EtOH	76.69	0.83	
127	*Rosa rugosa Thunberg.*	100% MeOH	50.19	4.26	
128	*Kalopanax pictus Nakai*	Hot water	120.17	0.42	
129	*Kalopanax pictus Nakai*	50% EtOH	117.01	1.70	
130	*Kalopanax pictus Nakai*	100% MeOH	71.50	2.07	
131	*Geranium Thunbergii*	Hot water	95.31	1.30	
132	*Geranium Thunbergii*	50% EtOH	107.77	1.24	
133	*Geranium Thunbergii*	100% MeOH	114.16	0.06	
134	*Fallopia japonica*	Hot water	105.51	0.52	
135	*Fallopia japonica*	50% EtOH	98.88	3.77	
136	*Fallopia japonica*	100% MeOH	112.49	1.07	
137	*Cudrania tricuspidata Bureau*	Hot water	108.66	0.95	
138	*Cudrania tricuspidata Bureau*	50% EtOH	114.63	0.28	
139	*Cudrania tricuspidata Bureau*	100% MeOH	98.95	0.32	
140	*Fagopyrum esculentum Moench / Polygonum emaginatum Roth*	Hot water	21.98	3.95	
141	*Fagopyrum esculentum Moench / Polygonum emaginatum Roth*	50% EtOH	84.45	1.93	
142	*Fagopyrum esculentum Moench / Polygonum emaginatum Roth*	100% MeOH	86.43	0.57	
143	*Agrimonia pilosa Ledeb. / Agrimonia dahurica (Link) Willd.*	Hot water	86.46	2.18	
144	*Agrimonia pilosa Ledeb. / Agrimonia dahurica (Link) Willd.*	50% EtOH	86.71	1.52	
145	*Agrimonia pilosa Ledeb. / Agrimonia dahurica (Link) Willd.*	100% MeOH	77.24	4.13	
146	*Aronia arbutifolia (L.) Pers.*	Hot water	92.80	2.40	
147	*Aronia arbutifolia (L.) Pers.*	50% EtOH	67.90	1.23	
148	*Hovenia dulcis Thunberg*	Hot water	46.68	2.36	
149	*Hovenia dulcis Thunberg*	50% EtOH	-4.95	3.17	
150	*Hovenia dulcis Thunberg*	Hot water	52.43	20.22	
151	*Hovenia dulcis Thunberg*	50% EtOH	126.48	1.01	○

Table S2. The list of 53 natural extracts for secondary screening (100 μg/mL) and their *P. falciparum* growth inhibition activity (%).

Rank	Plant species	Extraction Solvent	*P. falciparum* growth inhibition (%)	
			Mean	SD
1	*Geranium Thunbergii*	50% EtOH	114.90	0.14
2	*Geranium Thunbergii*	100% MeOH	110.20	1.70
3	*Fallopia japonica*	100% MeOH	94.25	12.23
4	*Platycodon grandiflorum*	50% EtOH	94.20	4.53
5	*Amomum villosum*	Hot water	93.75	1.34
6	*Cinnamomum zeylanicum Blume(leaf)*	Hot water	90.00	3.11
7	*Amomum villosum*	50% EtOH	84.15	0.92
8	*Fallopia japonica*	Hot water	65.25	0.07
9	*Cinnamomum zeylanicum Blume(leaf)*	50% EtOH	61.80	1.13
10	*Cinnamomum zeylanicum Blume(twig)*	Hot water	61.65	14.92
11	*Tussilago farfara L.*	Hot water	58.35	10.54
12	*Agastache rugosa O. Kuntze*	Hot water	55.60	6.93
13	*Albizzia julibrissin Durazz.*	100% MeOH	52.85	4.31
14	*Rosa laevigata Michaux*	Hot water	51.05	26.38
15	*Pueraria lobata Ohwi*	100% MeOH	43.30	11.60
16	*Eclipta prostrata L.*	50% EtOH	37.05	0.49
17	*Nelumbo nucifera Gaertner*	50% EtOH	37.00	1.56
18	*Agastache rugosa O. Kuntze*	50% EtOH	36.30	4.81
19	*Eclipta prostrata L.*	100% MeOH	35.40	3.82
20	*Echinops setifer Iljin*	Hot water	30.45	9.69
21	*Eclipta prostrata L.*	Hot water	28.05	0.35
22	*Angelica koreana L.*	Hot water	27.40	15.13
23	*Chrysanthemum indicum L.*	100% MeOH	23.65	2.33
24	*Vespae Nidus*	Hot water	23.65	0.78
25	*Prunella vulgaris L.*	100% MeOH	20.65	1.63
26	*Amomum villosum*	100% MeOH	19.25	5.30
27	*Kalopanax pictus Nakai*	Hot water	18.70	2.97
28	*Viscum album var. coloratum (Kom.) Ohwi*	Hot water	17.25	0.21
29	*Albizzia julibrissin Durazz.*	50% EtOH	16.90	0.42
30	*Pueraria lobata Ohwi*	50% EtOH	16.00	0.99
31	*Sophora flavescens*	50% EtOH	13.15	0.49
32	*Fallopia japonica*	50% EtOH	12.50	0.85
33	*Geranium Thunbergii*	Hot water	10.95	0.64
34	*Rosa rugosa Thunberg.*	Hot water	10.20	0.42
35	*Cibotium barometz J. Smith*	Hot water	9.95	7.42

36	*Albizzia julibrissin Durazz.*	Hot water	9.20	2.97
37	*Platycodon grandiflorum*	100% MeOH	8.75	7.14
38	*Lonicera japonica Thunberg*	50% EtOH	8.55	4.31
39	*Cudrania tricuspidata Bureau*	Hot water	8.45	1.63
40	*Cudrania tricuspidata Bureau*	50% EtOH	8.00	2.26
41	*Dianthus sinensis L.*	50% EtOH	7.85	0.21
42	*Kalopanax pictus Nakai*	50% EtOH	7.35	2.33
43	*Cirsium japonicum var. ussuriense (Regel) Kitam*	50% EtOH	7.00	2.40
44	*Gallus domesticus Brisson*	100% MeOH	4.70	3.11
45	*Cudrania tricuspidata Bureau*	100% MeOH	4.00	3.82
46	*Aronia arbutifolia (L.) Pers.*	Hot water	4.00	1.27
47	*Selaginella tamariscina Spring*	50% EtOH	2.75	20.01
48	*Tussilago farfara L.*	100% MeOH	2.10	1.98
49	*Cibotium barometz J. Smith*	50% EtOH	0.65	10.68
50	*Nelumbo nucifera Gaertner*	Hot water	-0.20	0.57
51	*Illicum verum*	50% EtOH	-0.55	2.47
52	*Glycyrrhiza uralensis Fischer*	100% MeOH	-2.80	0.99
53	*Sophora flavescens*	100% MeOH	-8.25	0.64

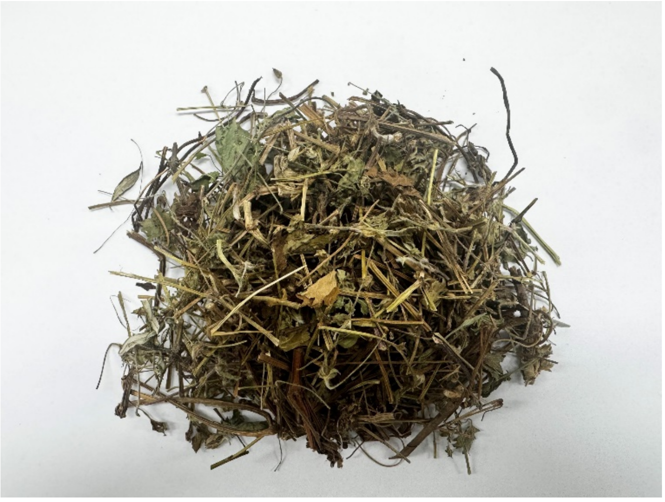

Figure S1. Dried *Geranium thunbergii* which is used for extraction.
